# An Analysis of the Putative CBD Binding Site in the Ionotropic Cannabinoid Receptors

**DOI:** 10.3389/fncel.2020.615811

**Published:** 2020-12-09

**Authors:** Chanté Muller, Patricia H. Reggio

**Affiliations:** Department of Chemistry and Biochemistry, University of North Carolina at Greensboro, Greensboro, NC, United States

**Keywords:** cannabidiol, TRP channels, TRPV1, TRPV2, TRPV3, TRPV4, TRPA1, TRPM8

## Abstract

Cannabinoids have been long studied for their therapeutic properties, particularly for their use in the treatment of pain. As new therapies are sought after to treat conditions of chronic pain, so is a better understanding of the ligands and their target receptors or channels. A recently published cryo-EM structure showed the putative binding location of a well-known cannabinoid ligand, cannabidiol (CBD), in TRPV2, a channel that has been implicated in inflammation and chronic pain. TRPV2, along with TRPV1, TRPV3, TRPV4, TRPA1, and TRPM8 all have the capability to be modulated by cannabinoid ligands and are located in the peripheral nervous system. Here, we analyze the putative CBD binding site in each of these channels and compare structural and sequential information with experimental data.

## Introduction

Transient receptor potential (TRP) ion channels are membrane-spanning channels that are formed by the homo- or hetero-tetramerization of TRP subunits. Each subunit contains six transmembrane helices (S1–S6), which, when tetramerized together, form a central pore, allowing for cation permeation (Levine and Alessandri-Haber, 2007). These channels, located in the plasma membrane, are capable of gating several mono- and di-valent cations through this pore in response to a stimulus. In mammals, six main subfamilies of TRP channels have been identified: ankyrin (TRPA), vanilloid (TRPV), melastatin (TRPM), canonical (TRPC), mucolipin (TRPML), and polycystin (TRPP) (Winter et al., 2013). Several of these channels have been implicated as sensors of many pathological and physiological processes including itch, temperature, genetic disorders, and pain related to cancers, AIDS, or other neuropathic conditions (Nilius and Vennekens, 2007; Vay et al., 2012; Perálvarez-Marín et al., 2013).

Chronic pain conditions remain a significant and prominent problem in today's society, effecting millions of people worldwide (Starkus et al., 2019). The complex mechanisms and etiologies that underlie chronic pain are diverse and cover a range of symptoms, conditions, and pathways (Levine and Alessandri-Haber, 2007) that can be brought on by a variety of causes including diabetes (Szallasi et al., 2007), stroke (Lau and Vaughan, 2014), and treatments for other conditions. Often, other symptoms like depression, anxiety, fatigue, and limitation of activity co-occur leading to an overall reduced quality of life (Ware et al., 2002). The current therapies to treat chronic pain conditions are considered to be relatively inadequate. NSAIDs, opioids, tricyclic antidepressants, local anesthetics, and antiepileptics can work to alleviate some chronic pain sufferers' experiences but don't often produce sustained relief (Levine and Alessandri-Haber, 2007; Luongo et al., 2017). With opioid medications, there are also dangers of dependence, tolerance, and addictive behaviors associated with their usage. In an effort to combat the overuse of opioid medications and resultant side effects, as well as find other meaningful therapies, different avenues of pain-related therapeutics are being investigated, notably the use of *Cannabis* in the treatment of chronic pain (Nielsen et al., 2017).

This perspective aims to discuss the putative binding site of a well-known cannabinoid, cannabidiol (CBD) in a selection of six TRP channels that are located in primary somatosensory neurons. This selection of channels (TRPV1–TRPV4, TRPA1, and TRPM8) have been identified as thermoTRPs, responding to various thresholds of temperature activation, as well as ionotropic cannabinoid receptors due to their ability to be modulated by cannabinoid ligands (Muller et al., 2019). The putative binding site of CBD has been identified in TRPV2 (Pumroy et al., 2019), and in an effort to better understand CBD interaction at the ionotropic cannabinoid receptors, a sequential and structural analysis will be discussed herein.

## Cannabinoids and the Modulation of Pain

*Cannabis* has been used for millennia to treat pain caused by various situations, including uses in ameliorating pain caused by childbirth in ancient Israel, as a surgical anesthetic in China, and for various painful ailments in the West in the 1800s (Walker and Huang, 2002; Ware et al., 2002). Today, one of the most commonly cited reasons for seeking medical marijuana is due to chronic pain (Ilgen et al., 2013; Klimkiewicz and Jasinska, 2018). There is extensive literature that supports the role of phytogenic and endogenous cannabinoid ligands as pain modulators (Maione et al., 2006; Lau and Vaughan, 2014), and the identification of broader targets for cannabinoid ligands also works to support this hypothesis (Caterina, 2014). Canonically, CB1 and CB2 are most widely known as receptors for cannabinoid ligands (Howlett, 2002; Pertwee et al., 2010; Morales et al., 2018). Pharmacological evidence shows, however, that other G protein-coupled receptors (GPCRs) (Morales et al., 2018) and other receptor types, including a subset of TRP channels, also have the ability to be modulated by cannabinoid ligands. Of the subfamilies of TRP channels found in mammals, at least three subfamilies contain channels that have been identified as having this ability, earning the name “ionotropic cannabinoid receptors” (Jordt et al., 2004; Akopian et al., 2009). Additionally, these channels, TRPV1–4, TRPA1, and TRPM8 can be found in primary somatosensory neurons, acting as sensory transducers that may participate in the generation of painful sensations evoked by thermal, mechanical, or chemical stimuli. These attributes make the ionotropic cannabinoid receptors a worthwhile target to investigate for the development of new pain therapies by targeting that which contributes to the detection of stimuli.

## Location, Location, Location

The location of the TRP channels assessed in this perspective covers many neuron types within the peripheral nervous system, ranging from small diameter peripheral sensory nerves (TRPM8) (Peier et al., 2002b), to small- to medium-diameter neurons (TRPV1) (Caterina et al., 1997; Tominaga et al., 1998; Sanchez et al., 2001), to medium- to large-diameter neurons that give rise to Aδ and Aβ fibers (TRPV2) (Caterina et al., 1999; Bender et al., 2005), to co-expression with TRPV1 (Story et al., 2003) in a subset of small- to medium-diameter neurons (TRPA1) (Bautista et al., 2005; Kobayashi et al., 2005; Caterina, 2006), and to predominant expression in keratinocytes (TRPV3 and TRPV4) (Peier et al., 2002a; Mandadi et al., 2009). These channels (TRPV1–4, TRPA1, and TRPM8), having a wide distribution throughout the peripheral nervous system, have been implicated in roles of conducting various sensations, some of which are familiar to many. The spiciness of chili peppers from capsaicin action at TRPV1, the pungency of garlic and wasabi produced by allicin and isothiocyanate modulation of TRPA1 (Jordt et al., 2004; Macpherson et al., 2005), and the cool, minty sensation of toothpastes or candies brought upon by menthol modulation of TRPM8 (Moran, 2018) are sought after as welcomed, albeit sometimes discomforting, sensations. However, in the case of chronic pain conditions, whether due to nerve injury or inflammation, ongoing painful stimulation leads to peripheral and central sensitization that can lead to painful sensations upon mild tactile stimulation (allodynia), greater than normal pain response to a stimulus (hyperalgesia), and spontaneous pain (Walker and Huang, 2002; Patapoutian et al., 2009).

## Differences in the Putative Cbd Binding Site of the Ionotropic Cannabinoid Receptors

**Figure 1:**
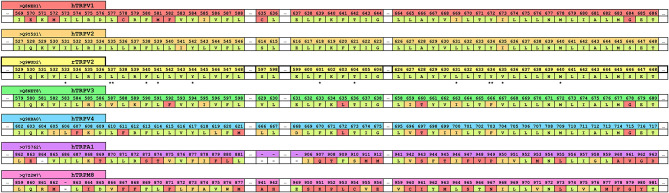
A truncated version of the human sequence alignment of six ionotropic cannabinoid receptors and rTRPV2. CBD has been resolved in rTRPV2 in two separate states and residues within 10 Å of the putative binding site of CBD have been highlighted and are shown here as a reference (yellow row). Comparable regions within the human ionotropic cannabinoid receptors have been aligned. Residues within hTRPV1 (red row), hTRPV2 (orange row), hTRPV3 (green row), hTRPV4 (blue row), hTRPA1 (purple row), and hTRPM8 (pink row) that are the same as the reference (rTRPV2) are shown in pale green. Residues that are of a similar type to the reference are shown in orange, and divergent residues are shown in red. The double starred residues, L537 and Y634, indicate the two residues that were noted to have rotameric changes from the apo to the CBD-bound structure of rTRPV2 and are visualized in Figure 2. Single starred residues are within 5 Å of bound CBD and are marked for easy vertical comparison across the ionotropic cannabinoid receptors.

**Figure 2:**
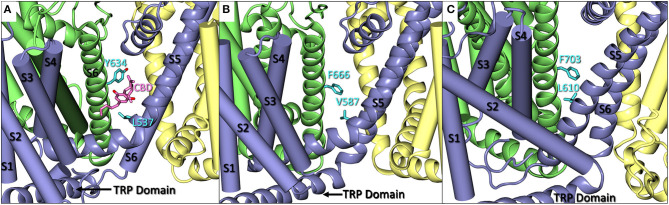
**(A)** A close up of CBD (pink) bound in rTRPV2 (adapted from PDB: 6U88) with Y634 and L537 in cyan. Helices S1–S4 and S6 are shown as cartoon tubes with S5, the pore helix, and the TRP domain shown as cartoon ribbons. **(B)** A close up of hTRPV3 (adapted from PDB: 6MHO) with comparable residues F666 and V587 shown in cyan. Helices S1–S4 are shown as cartoon tubes and S5-TRP domain are shown as cartoon ribbons. **(C)** A close up of *Xenopus tropicalis* TRPV4 (adapted from PDB: 6BBJ) which shares 78% sequence homology with human TRPV4. F703 and L610 are shown in cyan. Helices S1–S4 are shown as cartoon tubes, and S5-TRP domain are shown as cartoon ribbons.

Recently, a cryo-EM structure of rTRPV2 interacting with CBD has been elucidated in two separate states (Pumroy et al., 2019). The putative binding location of CBD was identified as the region between helix 6 of one monomer and helix 5 of the adjacent monomer (see Figure 2A), a general schematic of which can be found in the Supplementary Material. By using the information provided from the cryo-EM structures, we can identify regions of sequential homology and structural similarity in the putative CBD binding site across the ionotropic cannabinoid receptors. The sequence alignment (Figure 1) details the similarities between rTRPV2 and hTRPV2, as well as the human sequence of other ionotropic cannabinoid receptors TRPV1, TRPV3, TRPV4, TRPA1, and TRPM8. To get an idea of the binding site, residues within 10 Å of the putative CBD binding location in rTRPV2 these have been highlighted for analysis and directly compared to the other ionotropic cannabinoid receptors. A visual representation of the putative binding site(s) in three ionotropic cannabinoid receptors can be seen in Figures 2A–C.

### TRPV1

One of the most well-studied TRP channels is TRPV1, which shares an overall sequence identity of 50% with hTRPV2 (Bishnoi and Premkumar, 2013). TRPV1 is primarily known for its activation via vanilloid agonists, like capsaicin, and the subsequent desensitization it undergoes leading to the paradoxical analgesic effect elicited (Wong and Gavva, 2009). This effect is sought after for its potential use in pain therapies and is the basis of how some current topical-based therapies work, like Capzasin cream. TRPV1 shares the highest sequence identity within the putative CBD binding site as described above at 79%, second only to TRPV2 itself. Additionally, CBD is reported to have the highest efficacy of the vanilloid subfamily at TRPV1 (~78%) (Chianese et al., 2020).

Two residues that have been identified for their involvement of CBD binding in TRPV2, L535, and Y634 (Pumroy et al., 2019), can also be found in TRPV1 as L577 and Y672. L577 and Y672 are in comparable sequential and physical locations to their TRPV2 counterpart in addition to fairly consistent sequence homology about the rest of the binding site as seen in Figure 1. However, the tyrosine in either channel is unlikely to have a direct interaction with CBD in the putative binding site. Assuming the CBD binding site in TRPV1 is the same as that of TRPV2, this orientation of tyrosine would likely have minimal impact in the binding affinity of CBD, especially due to the reported role of Y634 in TRPV2 as a hydrophobic shield from water in the pore. Due to this, it is possible that Y672 in TRPV1 is still providing some degree of hydrophobic shielding for CBD should it bind in the same location, while the lipophilic interactions via L577 and surrounding lipophilic residues remain intact.

### TRPV2

TRPV2 is the thermoTRP with the highest temperature activation threshold among its subgroup with activation occurring above 52°C (Caterina et al., 1999). While TRPV2 is insensitive to capsaicin, it undergoes similar desensitization following activation and is deeply involved in inflammation and chronic pain (Levine and Alessandri-Haber, 2007). The cryo-EM structure of CBD in rTRPV2 was published in 2019 by Pumroy et al. and this structure allows us to delve deeper into how CBD interacts with TRPV2 specifically (Pumroy et al., 2019), but also hypothesize on the binding of CBD in other TRP channels that can be modulated by this cannabinoid ligand via sequential comparison and computational exploration.

A comparison of the rTRPV2 and hTRPV2 putative binding site of CBD shows 96% sequence homology with few instances of changes between hydrophobic residues, such as valine (rTRPV2) to isoleucine (hTRPV2). Beyond this, the polarity and aromaticity of residues within the binding site between rTRPV2 and hTRPV2 remains consistent (see Figure 1). Rotameric comparisons between the apo (PDB: 6U84 and 6U86) and CBD-bound (PDB: 6U8A and 6U88) cryo-EM structures for rTRPV2 revealed several rotameric changes throughout. Though most rotameric changes were peripheral and not within the defined scope of the CBD binding site, two rotameric changes were located within this scope. In the apo structure, Y634 takes on a g+ conformation while the CBD-bound structure shows Y634 adapting a trans conformation (Figure 2A). This movement shifts the hydroxyl group of the tyrosine toward the pore, believed to create some hydrophobic shielding for CBD from ions and water found there (Pumroy et al., 2019). Additionally, L537 shows a rotameric change, going from trans in the apo structure to g+ in the CBD-bound structure which is reported to allow accommodation for the aromatic ring of CBD. With two CBD-bound cryo-EM structures identifying CBD in the same general location (Pumroy et al., 2019) and a reported efficacy of ~67% at TRPV2 (Chianese et al., 2020), exploring this region should provide insight on the binding mode of CBD.

### TRPV3

TRPV3 is predominantly expressed in the brain (Xu et al., 2002) as well as several peripheral tissues like the skin and tongue (Wang et al., 2011). Additionally, TRPV3 acts as a thermosensor for innocuous warm temperatures, activating between 33 and 39°C (Szallasi et al., 2007). During a screen with a variety of phytocannabinoids, CBD was observed to have a potency similar to that of its typical agonist (De Petrocellis et al., 2012), carvacrol, though the efficacy of CBD at TRPV3 is poorer than that of TRPV1 and TRPV2 (~54 vs. ~78, ~67%, respectively). With 77% sequence homology in the CBD putative binding site, some small differences in sequence might be responsible for the low efficacy.

In TRPV2, Y634 is said to provide hydrophobic shielding for CBD by shifting to point the polar hydroxyl group toward the pore of the channel, preventing solvation of the location where CBD is proposed to bind. In TRPV3, this tyrosine is replaced by F666, losing the polar hydroxyl group which could lessen the shielding effect, but also occlude the binding site due to not being able to twist and point to the pore. Another sequential change that could impact the efficacy of CBD at TRPV3 is the change of leucine (L537, TRPV2) to valine (V587, TRPV3). While both hydrophobic, the shorter chain of the valine might affect the extent of the lipophilic interactions within TRPV3 (Figure 2B). While the efficacy of CBD at TRPV3 is generally considered poor, it still retains a submicromolar potency of around 0.51 μM (Chianese et al., 2020).

### TRPV4

The final vanilloid subfamily member discussed in this perspective is TRPV4. Similar to TRPV3, TRPV4 responds to warm thermal changes of temperatures ranging from 25 to 34°C (Vay et al., 2012). Additionally, this channel, located in cutaneous A and C fibers (Suzuki et al., 2003), plays a role in skin barrier function and nociception (Nilius and Owsianik, 2010).

Initially, it appears that there are only moderate differences in the putative CBD binding site in TRPV4 and TRPV2 due to the 68% sequence homology of this region. However, CBD is the least efficacious at TRPV4 with a mere 15% efficacy (Chianese et al., 2020). The aspect that likely has the largest effect on CBD binding is the structurally different helical arrangement that TRPV4 takes in comparison to its family members. TRP channels in this subgroup typically adopt a “straddling” formation of S1–S4 over the TRP domain where S1 and S4 reside on one side of the TRP domain and S2 and S3 reside on the other. TRPV4, however, does not appear to follow suit. In a series of recently published cryo-EM structures of *Xenopus tropicalis* TRPV4, which maintains 78% sequence homology with hTRPV4 (Deng et al., 2018), helical packing of S1–S4 against S5–S6 was observed to be different from that of other resolved TRPV channels, such as TRPV1, TRPV2, and TRPV3 (Deng et al., 2018). Helices S1, S3, and S4 all appear on one side of the TRP domain, leaving S2 on the other. This differentiation in helical arrangement, as well as the angle at which S2 takes on in TRPV4, alters the shape of the S1–S4 bundle (see Figure 2C), affecting the putative CBD binding site by altering the interaction between S5 and S6 of adjacent monomers. Deng et al. note that this unique S1–S4 packing arrangement may be due to truncation of TRPV4, though it is reported that only the unstructured N- and C-termini were truncated. Additionally, multiple TRPV4 structures with various cations were resolved, all maintaining this feature. The S4 helix obstructs the putative CBD binding site located in this channel, lending support that this strange arrangement of S1–S4 helices is plausible rather than an artifact of truncation and/or crystallization.

### TRPA1

The first member of the ankyrin subfamily, TRPA1, can be found co-expressed with TRPV1 in a subset of peripheral sensory neurons (Patapoutian et al., 2009). TRPA1 is activated by isothiocyanates, pungent compounds found in mustard, garlic, and onions (Niforatos et al., 2007), covalently binding to an internal cysteine or lysine residue located on its extensive ankyrin repeat domain. With regard to its role as a thermoTRP, TRPA1 can be found on the lowest end of the temperature scale, activating below temperatures of 17°C (Story et al., 2003). Additionally, TRPA1 plays an important role in neuropathic and inflammatory pain through the mediation of bradykinin-evoked and mechanical hyperalgesia (Jordt et al., 2004).

CBD has been shown to act as an agonist at TRPA1 with an efficacy of 108% compared to its usual agonist of allylisothiocyanate (100 μM) (Chianese et al., 2020), and while the putative CBD binding site is structurally comparable to that of the TRPVs, there is low sequence homology within the binding site (~30%). Similar to the previous TRP channels discussed, TRPA1 maintains a leucine in the same position as in TRPV2 (L870 and L537, respectively). Looking at the sequence alignment (Figure 1), hydrophobic residues are readily present and are, in fact, pointing in the region where CBD is proposed to bind. However, since the efficacy of CBD at TRPA1 is considerably higher than TRPV2, the sequence homology with TRPV2 might be of little importance in this case.

### TRPM8

Finally, the last member of the ionotropic cannabinoid receptors that will be discussed here is TRPM8. TRPM8 is activated by temperatures below 27°C (de la Peña et al., 2005) as well as by compounds that elicit a “cooling” effect, such as menthol, eucalyptol, and icilin (McKemy et al., 2002; Peier et al., 2002b; Chuang et al., 2004). Compounds tested at TRPM8 are usually tested for their antagonism against both menthol and icilin as they are reported to activate the channel in slightly different locations (Chuang et al., 2004; Bandell et al., 2006; Yin et al., 2018; Xu et al., 2020). For both ligands, CBD acts as an antagonist at submicromolar concentrations (De Petrocellis et al., 2008).

Because of this, it is slightly more difficult to hypothesize if CBD binds in the same location in TRPM8 as it would in the TRPV or TRPA subfamilies. The binding site in TRPM8 has ~30% sequence homology with that of TRPV2, but because CBD acts as an antagonist, the location of binding as well as the movements required by the channel to become inactive, could require different mechanisms than activation of the other ionotropic cannabinoid receptors.

## Concluding Remarks and Future Directions

Targeting the endocannabinoid system has been a promising strategy for the modulation of pain (Maione et al., 2006; Lau and Vaughan, 2014; Aizpurua-Olaizola et al., 2017; Chanda et al., 2019). One in particular, CBD, has gained mainstream attention in recent years due to over the counter (OTC) uses in balms, creams, tinctures, and more for joint and muscle pain, neuroprotection, anti-nausea, anti-inflammation, and anxiolytic properties (Mechoulam et al., 2007), as well as pharmaceutical uses in drugs, such as Sativex, a 1:1 CBD:THC oromucosal spray has been approved for use in the UK to aid in the relief of multiple sclerosis (MS) related symptoms, and Epidiolex, an FDA-approved CBD-based drug used to treat two severe forms of pediatric epilepsy. Additionally, there is literature precedent to support the claims of CBD as a means to treat other conditions, such as arthritis (Malfait et al., 2000), anxiety (Guimaraes et al., 1994), and the potential for treatment in substance use disorders (Wiese and Wilson-Poe, 2018; Turna et al., 2019). By seeking to understand the targets of cannabinoid ligands, and particularly where they bind, researchers will be better equipped to design drugs to treat chronic pain disorders.

Recently, a dimerized version of CBD (CBDD or cannabitwinol) has been isolated, structurally characterized, and tested by Chianese et al. at the ionotropic cannabinoid receptors (Chianese et al., 2020). What was discovered was that CBDD was found to be nearly inactive at TRPV1 and TRPV2, in contrast to the good efficacies of CBD at these two channels, while exhibiting poor efficacy at TRPV3 and TRPV4, and retaining activity as a TRPA1 activator (~97% efficacy) and TRPM8 inhibitor against icilin (IC_50_ = 3.9 ± 0.4 μM). One structural factor that may play a role in how well CBDD interacts with TRPA1, despite its doubling in size, is the TRP-like domain which lies lower in the intracellular region than a TRP domain (Paulsen et al., 2015). The TRP domain found in TRPV1–4 and TRPM8 is nestled just below the lower leaflet in the intracellular region of the cell. In TRPV2, the S1–S4 domain straddles the TRP domain which acts as a “floor” to the putative CBD binding site. Since this feature is not present in TRPA1, instead having a TRP-like domain, more space is created in the putative binding region, potentially allowing for easier access by CBD and its dimerized sibling. If we hypothesize that CBDD binds in the same location as CBD in TRPA1, it is sensible to think that the increase in room from the TRP-like domain would allow more space for CBDD to fit, even if one-half of dimerized CBD were to bind, potentially leaving the other half “left out” of the binding site. Conversely, based on the features discussed here, CBDD would have limited space in TRPV1–TRPV4.

The comparison of the putative CBD binding site presented here is based on the sequence alignment of the ionotropic cannabinoid receptors and previously published crystal or cryo-EM structures from a molecular modeling perspective. Ideally, future work would combine cryo-EM/crystal structures of CBD interacting with the other TRP channels in addition to site-directed mutagenesis to further investigate these interactions.

## Data Availability Statement

The original contributions presented in the study are included in the article/Supplementary Material, further inquiries can be directed to the corresponding author/s.

## Author Contributions

CM wrote the manuscript and prepared the figures with guidance and editing from PR. All authors contributed to the article and approved the submitted version.

## Conflict of Interest

The authors declare that the research was conducted in the absence of any commercial or financial relationships that could be construed as a potential conflict of interest.
