# Supplementary material for: An Analysis of the Putative CBD Binding Site in the Ionotropic Cannabinoid Receptors
Source: Front Cell Neurosci. 2020 Dec 9;14:615811. doi: 10.3389/fncel.2020.615811 (PMC7755602; doi:10.3389/fncel.2020.615811)
Supplement: Supplementary file 1 [file Table_1.docx]

Supplemental Figure 1.

Panel A) A cartoon image of one monomer of a TRP channel. S1 is shown in red, S2 in orange, S3 in yellow, S4 in light green, the S4-5 linker in dark green, S5 in light blue, S6 in dark blue, and the TRP domain (present in TRPV1-4 and TRPM8, comparable to TRP-like domain present in TRPA1). Panel B displays the same monomer with CBD, shown as a pink oval, in one-half of the putative binding site. Panel C displays a second monomer, with the same color-coding as panels A and B, pivoted at a 90°-degree angle to complete the putative binding site of CBD with S6 of the first monomer (Panel B) shown “behind” the CBD molecule and S5 of the second monomer (Panel C) shown “in front” of the CBD molecule, forming the putative binding site.
