# Supplementary figures and images for: An Analysis of the Putative CBD Binding Site in the Ionotropic Cannabinoid Receptors
Source: Front Cell Neurosci. 2020 Dec 9;14:615811. doi: 10.3389/fncel.2020.615811 (PMC7755602; doi:10.3389/fncel.2020.615811)

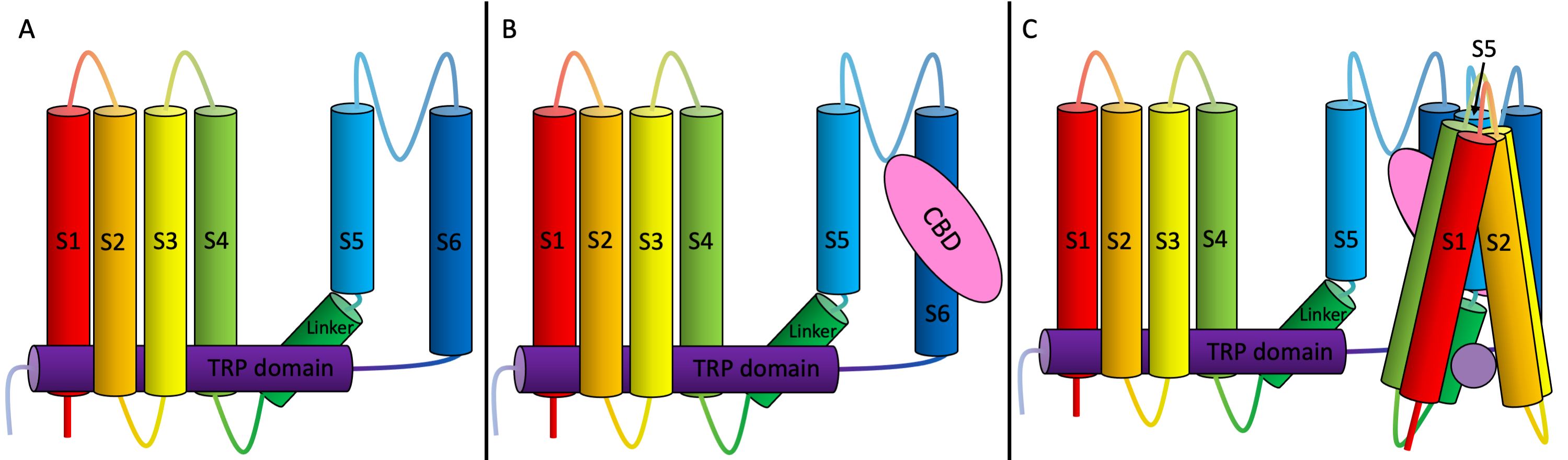

Supplement: Supplementary file 2 [file Image_1.TIFF]
